# Abnormalities in Electroencephalographic Microstates Among Adolescents With First Episode Major Depressive Disorder

**DOI:** 10.3389/fpsyt.2021.775156

**Published:** 2021-12-17

**Authors:** Yuqiong He, Qianting Yu, Tingyu Yang, Yaru Zhang, Kun Zhang, Xingyue Jin, Shuxian Wu, Xueping Gao, Chunxiang Huang, Xilong Cui, Xuerong Luo

**Affiliations:** ^1^National Clinical Research Center for Mental Disorders, Department of Psychiatry, The Second Xiangya Hospital of Central South University, Changsha, China; ^2^Autism Center of the Second Xiangya Hospital, Central South University, Changsha, China

**Keywords:** adolescents, major depressive disorder, EEG resting state, EEG microstate, first-episode depression

## Abstract

**Background:** Recent studies have reported changes in the electroencephalograms (EEG) of patients with major depressive disorder (MDD). However, little research has explored EEG differences between adolescents with MDD and healthy controls, particularly EEG microstates differences. The aim of the current study was to characterize EEG microstate activity in adolescents with MDD and healthy controls (HCs).

**Methods:** A total of 35 adolescents with MDD and 35 HCs were recruited in this study. The depressive symptoms were assessed by Hamilton Depression Scale (HAMD) and Children's Depression Inventory (CDI), and the anxiety symptoms were assessed by Chinese version of DSM-5 Level 2-Anxiety-Child scale. A 64-channel EEG was recorded for 5 min (eye closed, resting-state) and analyzed using microstate analysis. Microstate properties were compared between groups and correlated with patients' depression scores.

**Results:** We found increased occurrence and contribution of microstate B in MDD patients compared to HCs, and decreased occurrence and contribution of microstate D in MDD patients compared to HCs. While no significant correlation between depression severity (HAMD score) and the microstate metrics (occurrence and contribution of microstate B and D) differing between MDD adolescents and HCs was found.

**Conclusions:** Adolescents with MDD showed microstate B and microstate D changes. The obtained results may deepen our understanding of dynamic EEG changes among adolescents with MDD and provide some evidence of changes in brain development in adolescents with MDD.

## Introduction

Major depressive disorder (MDD), characterized by low mood, loss of interest, possible symptoms of physical discomfort, suicidal behaviors, and cognitive dysfunction, is one of the most common mental disorders (1), and has been the second leading cause of disability worldwide because of its significant impact on the quality of life (2). In recent years, the prevalence of MDD has increased in adolescents and young adults (3), and up to 20% of adolescents are affected by MDD, which severely affected their lives and increased the risk of suicide (4). Early diagnosis and treatment contribute to decreasing MDD severity and improving the well-being and prognosis of MDD patients. Due to lack of effective and specific indicators, the diagnosis of MDD is based on patients' clinical symptoms, which is more tricky for adolescents as the manifestation of MDD among them is less typical than among adults (5, 6). Therefore, identifying potential biomarkers of depression is very important for the diagnosis and treatment of MDD, and studies on MDD among adolescent population are necessary and urgent.

A large number of functional magnetic resonance imaging (fMRI) studies have reported changes in brain structure and function among MDD patients (7, 8), including reduced hippocampal volume (9), thinner cortex in parahippocampal-limbic and insular-limbic areas (10), reduced connectivity within the frontoparietal control system (11) and disrupted network connectivity in the default mode network (DMN) (12, 13). Although many fMRI studies have explored differences between MDD patients and healthy subjects in depth, these studies cannot dynamically assess changes of brain with time. The electroencephalograms (EEG) can compensate for this defect of fMRI because of its good time resolution and can capture rapid changes in the dynamics of neuronal network (14). Because of its low cost, non-invasive, and easy to complete, EEG has been widely used in the neurocognitive disciplines in recent years. Previous studies among patients with MDD have found higher theta power in the frontal cortex and rostral anterior cingulate compared to healthy subjects (15). Higher power of the gamma band was also found among adult MDD patients with suicide ideation (16). Researches among adolescents with MDD showed lower theta band and decreased resting-state connectivity in the frontal cortex (17). Lower left-sided alpha power was also found in adolescents with MDD, and left-sided alpha power was related to depression scores (18). Currently, all of these EEG analyses are mainly based on traditional approaches, such as powder spectral analysis (18) and resting-state connectivity (17).

Resting EEG involves a limited number of potential topographic maps, as each topographic map remains stable for a certain period of time (60–120 ms) before quickly switching to another topographic map. All these topographic map are called “EEG microstates,” and they dynamically change with time in an organized manner (19). The change in the EEG microstate indicates a change in the overall coordination of neuronal activity in the participant over time (20). Although different numbers of cluster maps have been reported in previous study (21, 22), four cluster maps, which are termed A, B, C, and D, are consistently identified in majority researches (23, 24). Studies based on EEG-fMRI technical have revealed the hemodynamic correlation of EEG microstate: microstate A may be associated with auditory network, while microstate B may be associated with the visual network, microstate C may reflect activity in the default mode network and the activity of the dorsal attention network may be related to microstate D (20, 25). Based on the dynamic changes of the four EEG microstate maps, the spontaneous brain activity can be represented by the duration, occurrence, contribution and transition possibility sequence of each EEG microstate maps (20). Capturing the temporal difference of dynamic changes of EEG microstates could be a promising method to study the spontaneous brain activity of adolescents with MDD.

In recent years, EEG microstate analysis has been used in patients with mental disorder, such as bipolar disorder (26), methamphetamine use disorder (27), schizophrenia (28), and depression (21, 22). Different psychopathological conditions showed different EEG microstate feature changes. Microstate B features were found to be related to depression scores among bipolar disorder patients (26). Studies among adults with MDD found that higher occurrence of microstate A was associated with depressive symptoms (21), and the contribution and duration of microstate D were reduced among depressed patients compared to healthy subjects and related to depression severity (22). Existing studies also found that EEG microstate feature may change with medication or the use of repetitive transcranial magnetic stimulation (rTMS). The duration, occurrence and contribution of microstate B decreased among the MDD patients after 2 weeks medication (29). These findings indicate that in-depth study of EEG microstates may provide new evidence for the diagnosis and treatment of adolescents with MDD. While to date, there has been no research on EEG microstate among MDD adolescents.

Here, we used EEG microstate as a new approach to explore the difference of spontaneous resting EEG between adolescents with MDD and HCs. The aims of the present study were to test whether there are differences in the temporal characteristics of EEG microstates between adolescents with MDD and HCs and whether there is a correlation between clinical symptoms and microstates.

## Materials and Methods

### Participants

The MDD patients were recruited in the outpatient clinic of the Second Xiangya Hospital, Central South University. The diagnostic process was performed by a child and adolescent psychiatrist with an intermediate professional title or above. The kid version of the Mini International Neuropsychiatric interview (Mini-kid) was used to confirm the diagnosis and to eliminate other mental disorders. The inclusion criteria for patients were: (1) 12–17 years old; (2) a diagnosis of MDD according to the Diagnostic and Statistical Manual of Mental Disorder-Fifth Edition criteria (DSM-5); (3) first episode; (4) no history of psychiatric drug treatment before completing the examination; (5) the Children's Depression Inventory (CDI) score ≥19 (30) and Hamilton Rating Scale for Depression (HAMD-17) score ≥17. The Mini-kid and the assessment for the depression symptoms (HAMD-17) were completed by two professional psychiatrists, who received consistent training before the beginning of the experiment. The HCs were recruited through advertising in a junior school in Changsha, Hunan Province and in online social media platforms (WeChat Moments). The inclusion criteria for the HCs were: (1) 12–17 years old; (2) no history of psychiatric illness by the Mini-kid interview; (3) no first-degree relatives with psychiatric disorders; (4) no history of psychiatric medication use. All the subjects were interviewed by two professional psychiatrists and met the inclusion criteria. Those subjects (1) meeting the diagnostic criteria for mental disorders other than depression (only for MDD patients); (2) suffering from other nervous system diseases (brain trauma, epilepsy, intracranial tumors, etc.) or serious physical diseases; or (3) unable to complete the examination for other reasons were excluded. Finally, 35 adolescents with MDD and 35 HCs participated this study. The general information such as gender, age and education years were collected among both groups, and the duration of illness were collected among the patient group.

This study was approved by the National Clinical Center Medical Ethics Committee of the Second Xiangya Hospital, Central South University. Written informed consent was provided by both the participants and their guardians.

### Symptom Ratings

Depression symptoms were assessed using the Children's Depression Inventory (CDI) (31) and Hamilton Rating Scale for Depression (HAMD-17). CDI is the most widely used self-assessment scale for children and adolescents with MDD. The CDI consists of 27 items scored using 0–2 points. The total score is obtained by totaling all items: the higher the score, the higher the degree of depression. Cronbach's α for the CDI was found to be 0.8504 in a study of Chinese children and adolescents (32). The HAMD-17 was conducted as an interview with the subjects to assess the severity of depressive symptoms. HAMD-17 consists of 17 items scored on a 5-point scale, with 0 indicating no symptoms and 4 indicating extremely severe symptoms. Anxiety symptoms were assessed using the Chinese version of the DSM-5 Level 2-Anxiety-Child scale. It consists of 13 items scored on a scale of 1–5: the higher the score, the higher the anxiety symptoms. Cronbach's α for this test was 0.90 in a study of Chinese children and adolescents (33).

### EEG Recordings and Pre-processing

A standard clinical EEG protocol of 5 min (resting-state and eyes closed) was recorded using 64 scalp electrodes with the International 10–20 system. Participants were asked to seat in a quiet room comfortably. The electrooculogram was recorded by one facial electrode located 1 cm below the middle of the right eye. EEG data were recorded at a sampling rate of 5,000 Hz. The data were referenced to the FCz electrode. Before the experiment, we reduced the electrode impedance below 5K Ω.

Pre-processing was performed using MATLAB 2013b and EEG tools. First, the sampling rate was lowered to 500. The data were band-pass filtered with cutoffs of 0.1–45 Hz and segmented into 2 s per epoch. When there were artifacts in a channel, the spherical interpolation method was used for interpolation (34), and <6 channels for each subject were replaced. When the signal quality of a segment was poor (the voltages of more than 10 channels exceed 80 mV), it would be excluded. After that, each subject retained at least 3 min EEG signals. Eye artifact correction was performed using independent component analysis (35).

### Microstate Analysis

The Microstate Analysis plug-in (Version 0.3) for EEGLAB (34) developed by Thomas Koenig was used for the microstate analysis. Artifact-free EEGs were band-pass filtered between 2 and 20 Hz then the data were re-referenced for the whole brain (36). The global field power (GFP) is calculated, and topographic maps selected for clustering are those at GFP peak (37). The polarity of the clusters was ignored. The number of clusters was selected as four based on previous researches which four clusters have been widely used and differences were found between HCs and patients with mental disorders (24). The microstate map of each participant was calculated by the original instantaneous diagram using atomic aggregation hierarchical clustering (AAHC) (38). The grand-mean model diagrams of each group (HC group, MDD group) were calculated and labeled as “A,” “B,” “C,” and “D.” The first and last segments were ignored by the microstate toolbox. The following parameters were extracted from the microstate data: duration (average duration of the four different microstate categories), occurrence (how many times per second the four microstate categories appear on average), contribution (percentage of time covered by the four microstate categories), and transition possibility (probability of conversion between the four microstate categories). The global explained variance (GEV) is calculated to assess to what extent the microstate topographic can explain the original EEG data.

### Data Analysis

Data were analyzed using SPSS version 21 (IBM). Categorical variables are reported as the count (*n*) and percentage (%). Continuous variables are expressed as the mean (*M*) ± standard deviation (SD). The independent sample *t*-test was used to compare general demographic information between the two groups. We used 4 (microstate classes) × 2 (groups) repeated measures analysis of variance (ANOVA) to evaluate the interaction effects of each microstate parameter. Greenhouse–Geisser correction for ANOVA was applied. The two tailed unpaired *t*-test was performed for each microstate separately as *post-hoc* analysis to assess the difference between MDD patients and HCs, if the interaction microstate ^*^ group was significant. Bonferroni correction was used for the *post-hoc* comparisons, and *p* < 0.013 (i.e., 0.05/4, there were 4 different types of microstates) was considered statistically significant for microstate features. Pearson correlation analysis was used to analyze the relationships between HAMD scores and the microstate metrics differing between MDD adolescents and HCs. The statistical tests were two-sided and the level of statistical significance was set as α = 0.05. Bonferroni correction was applied for multiple correlation analyses.

## Results

### Participant Characteristics

**Figure 1:**
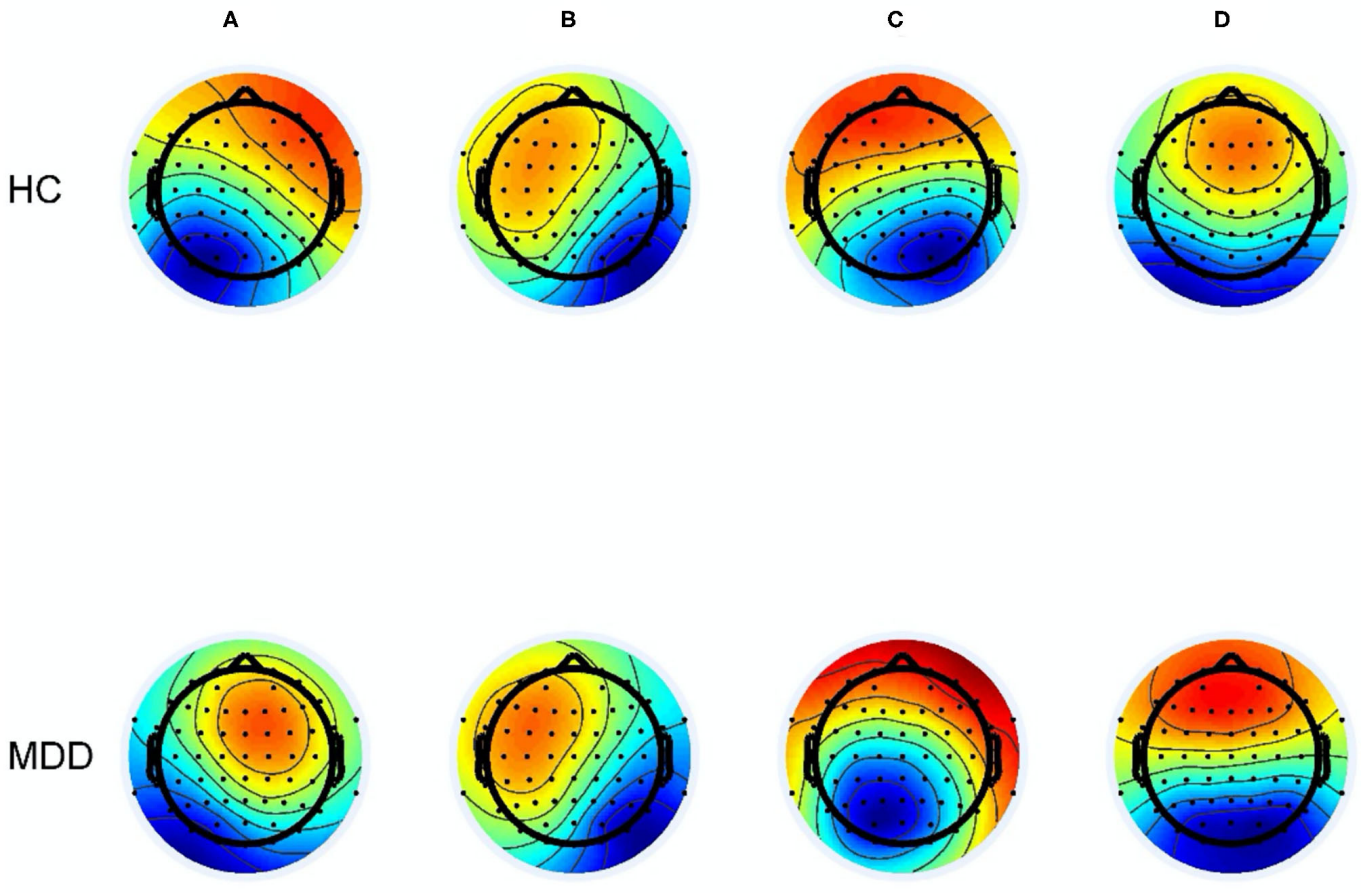
Mean scalp topographies of microstates **(A–D)**.

The demographic characteristics of the two groups and symptom ratings in the MDD group are shown in Table 1. There were no significant differences in age, education years, or gender between the two groups (all *p* > 0.05). For patients with MDD, the mean duration of illness was 15.20 ± 11.07 months. The CDI score of the MDD group was 38.34 ± 7.85, higher than that in HCs (9.57 ± 4.65), and the difference was significant (*p* < 0.001). The anxiety score was 40.26 ± 7.85, and the HAMD score was 21.97 ± 2.73 among the MDD group. Four microstates (classes A to D) in the two groups were identified by AAHC. The average global explained variance was 74.08 ± 1.92% and 73.77 ± 2.74% for MDD group and HCs, respectively, and the difference was not significant (*p* = 0.579). The four microstate topographic maps resemble those in the previous literature in both groups (Figure 1).

**Table 1:** Demographic and clinical data of the participants.

**Characteristic**	**MDD**	**HC**		
	***N* = 35**	***N* = 35**	***t*/χ^2^**	***p***
Age, *M* (SD)	14.58 ± 1.46	15.05 ± 1.67	−1.241	0.219
Gender, male/female (*n*)	10/25	15/20	1.556	0.212
Education years, *M* (SD)	8.69 ± 1.47	8.69 ± 1.71	0.251	1
Duration of illness (months)	15.20 ± 11.07	NA	NA	NA
CDI score, *M* (SD)	38.34 ± 7.85	9.57 ± 4.65	18.662	<0.001
Anxiety score, *M* (SD)	40.26 ± 7.85	NA	NA	NA
HAMD score, *M* (SD)	21.97 ± 2.73	NA	NA	NA
GEV (%)	74.08 ± 1.92	73.77 ± 2.74	0.558	0.579

*M, mean; SD, standard deviation; MDD, major depressive disorder; HC, healthy control; GEV, global explained variance*.

### Microstate Metrics

**Figure 2:**
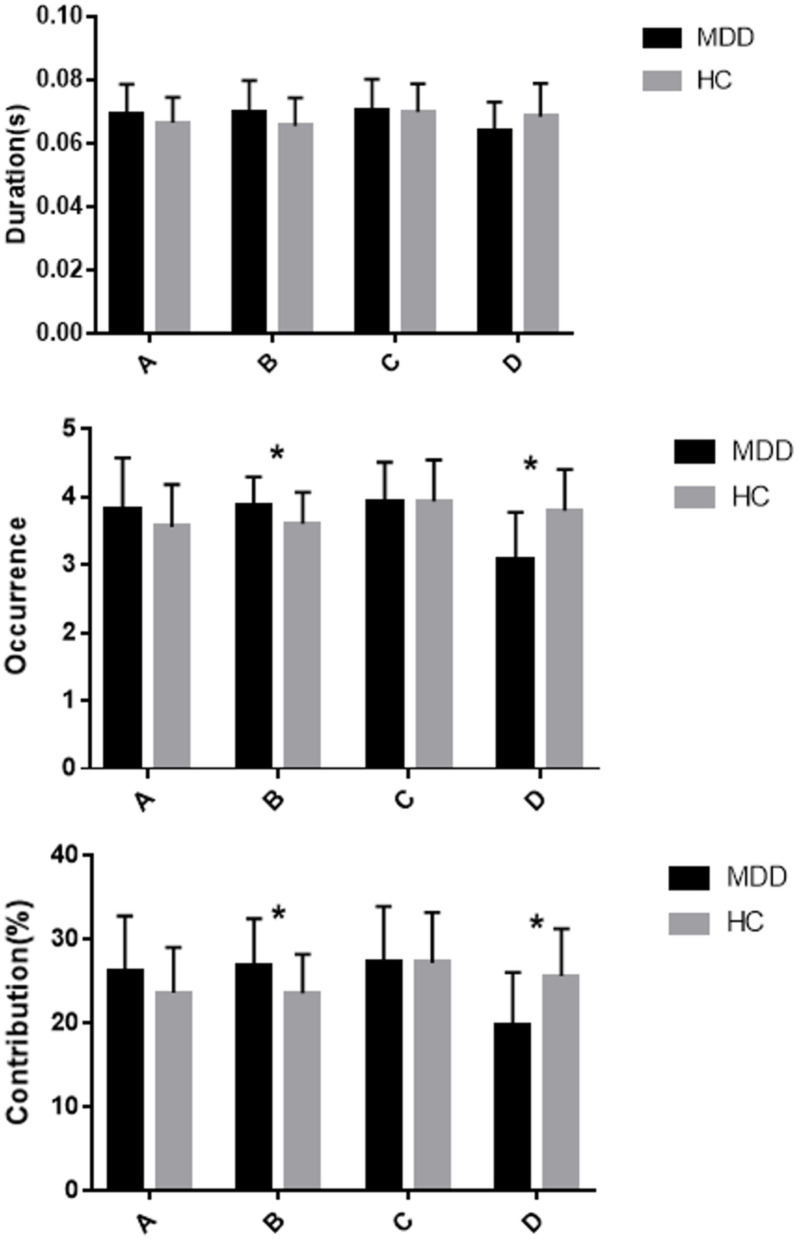
Mean and standard error of microstate metrics (duration, occurrence, and contribution) of MDD (black) and HC (gray) groups * significant difference between MDD and HC groups, as assessed by the unpaired *t*-test with Bonferroni correction.

Repeated measures ANOVA was conducted to compare the microstate duration, occurrence, contribution and transition possibility between the two groups. EEG microstate (A, B, C, D) were regarded as a within-subject factor and group (MDD or HC) as a between-subject factor. The results for microstate duration showed that the Microstate ^*^ Group interaction effect was significant (*F* =4.559; *p* = 0.006), while the within-subject factor effect (*F* =1.657; *p* = 0.185) and group effect (*F* = 0.373; *p* = 0.543) were not significant. *Post-hoc* comparisons showed no significant difference in microstates (A, B, C, D) between the two groups (all *p* > 0.013; Figure 2; Table 2).

**Table 2:** Comparison of microstates between the MDD group and HC group.

		**MDD**		**HC**			
		***M***	**SD**	***M***	**SD**	***t***	***p***
**Duration**	A	0.0694	0.0094	0.0667	0.0080	1.266	0.210
	B	0.0700	0.0100	0.0656	0.0089	1.921	0.059
	C	0.0705	0.0100	0.0700	0.0089	0.180	0.858
	D	0.0643	0.0089	0.0685	0.0106	−1.800	0.076
**Occurrence**	A	3.8142	0.7684	3.5605	0.6258	1.512	0.135
	B	3.8897	0.4083	3.6105	0.4615	2.681	0.009
	C	3.9352	0.5801	3.9337	0.6130	0.011	0.992
	D	3.0691	0.7062	3.7987	0.6058	−4.639	<0.001
**Contribution**	A	0.2617	0.0663	0.2366	0.0540	1.807	0.075
	B	0.2686	0.0561	0.2359	0.0462	2.657	0.010
	C	0.2721	0.0673	0.2722	0.0604	−0.001	0.999
	D	0.1976	0.0632	0.2564	0.0564	−4.107	<0.001

*M, mean; SD, standard deviation; MDD, major depressive disorder; HC, healthy control. Bonferroni correction for the post-hoc comparisons: p < 0.013*.

The results for microstate occurrence showed there were significant main effect of microstate (*F* = 9.827; *p* < 0.001) and Microstate ^*^ Group interaction effect (*F* = 9.606; *p* < 0.001), but no significant main effect of Group (*F* = 0.479; *p* = 0.491). *Post-hoc* analysis showed that the MDD group had a higher frequency of microstate B and a lower frequency of microstate D compared to the HC group, and these differences were statistically significant (both *p* < 0.013). The occurrence of microstates A and C did not differ significantly between the MDD group and HC group (*p* = 0.135 and *p* = 0.992, respectively; Figure 2; Table 2).

For the contribution of microstates, the main effect of microstate (*F* = 5.260; *p* = 0.003) and Microstate ^*^ Group interaction effect (*F* = 7.889, *p* < 0.001) were significant, but the main effect of Group (*F* = 0; *p* = 1) was not significant. *Post-hoc* analysis showed more microstate B contribution and less microstate D contribution in the MDD group compared to the HC group, and the differences were statistically significant (both *p* < 0.013). There were no statistically significant differences in other microstate contributions between the HC and MDD groups (both *p* > 0.013; Figure 2; Table 2).

For the transition probabilities, there were significant main effect of the microstate (*F* = 3.878; *p* < 0.001) and significant Microstate ^*^ Group interaction effect (*F* = 5.174, *p* < 0.001), while the main effect of Group (*F* = 0.582; *p* = 0.448) was not significant. *Post-hoc* analysis showed that the MDD group had a lower possibility of transition from “A to D,” “C to D,” “D to A,” and “D to C” than the HC group, and the differences were significant (all *p* < 0.013). Furthermore, the MDD group showed a higher possibility of transition from “A to B,” “A to C,” “B to A,” and “C to B” than the HC group, and the differences were significant (all *p* < 0.013; Table 3).

**Table 3:** Comparison and means for all transition probabilities in the MDD and HC groups.

	**MDD**		**HC**				
	**Mean**	**SD**	**Mean**	**SD**	***t***	***p***	**Direction**
A-B	0.0910	0.0246	0.0721	0.0179	3.662	<0.001	MDD > HC
A-C	0.0953	0.0265	0.0786	0.0209	2.936	0.005	MDD > HC
A-D	0.0619	0.0181	0.0779	0.0153	−3.999	<0.001	MDD < HC
B-A	0.0920	0.0227	0.0721	0.0187	3.994	<0.001	MDD > HC
B-C	0.0942	0.0209	0.0841	0.0181	2.157	0.035	MDD > HC
B-D	0.0689	0.0277	0.0768	0.0225	−1.311	0.194	
C-A	0.0940	0.0263	0.0812	0.0205	2.270	0.026	MDD > HC
C-B	0.0946	0.0197	0.0831	0.0166	2.626	0.011	MDD > HC
C-D	0.0688	0.0204	0.0902	0.0237	−4.055	<0.001	MDD < HC
D-A	0.0622	0.0168	0.0751	0.0164	−3.267	0.002	MDD < HC
D-B	0.0697	0.0278	0.0778	0.0224	−1.337	0.186	
D-C	0.0678	0.0216	0.0918	0.0252	−4.284	<0.001	MDD < HC

*M, mean; SD, standard deviation; MDD, major depressive disorder; HC, healthy control. Bonferroni correction for the post-hoc comparisons: p < 0.013*.

### Relationship Between Microstate Metrics and Depression Score

Finally, we examined the relationship between depression severity (HAMD score) and the microstate metrics (occurrence and contribution of microstate B and D) differing between MDD adolescents and HCs. We found that contribution of microstate B is correlated with HAMD score (*r* = 0.341, *p* = 0.045, uncorrected). After Bonferroni correction was applied, the significance was gone (*p* > 0.013, Table 4).

**Table 4:** Correlation for microstate metrics and HAMD score.

	***r***	***p***
Occurrence B	0.253	0.143
Occurrence D	0.017	0.923
Contribution B	0.341	0.045
Contribution D	0.089	0.611

*Bonferroni correction for multiple correlations: p < 0.013*.

## Discussion

To the best of our knowledge, this is the first study to explore the dynamic activity of resting-state large-scale brain networks among adolescents with MDD. Our results indicate that adolescents with MDD show alterations in sub second of the whole brain. Compared to HCs, adolescents with MDD showed abnormally increased occurrence of microstate B and decreased occurrence of microstate D. The increase in microstate B may be caused by more transitions from microstate C to microstate B. Adolescents with MDD had less transitions from “A to D” and more transition from “D to C,” which were the reasons for the decrease in microstate D among MDD subjects compared with the HCs.

Limited research has examined EEG microstate duration and/or occurrence in depressed patients. Our results showed the difference in microstate duration between adolescents with MDD and HCs was not obvious, which is inconsistent with other studies in adult MDD patients (22, 39). For example, Murphy et al.'s (22) research showed that the duration of microstate D was reduced among the adults MDD patients compared to the healthy subjects. However, our results are in line with the Damborská's results which found no difference in duration between the adults MDD patients and HCs (21). Although there was no change in the duration, the occurrence and contribution changed in adolescents with MDD. Our results showed that adolescents with MDD patients had higher contribution, occurrence of microstate B. This finding is in line with a previous research which found that the duration, occurrence, and contribution of microstate B were decreased as depressive symptoms improved among MDD adults (29). The head surface signal source displays microstate B was closely associated with right posterior alpha activity by accurate low resolution electromagnetic tomography (eloreta) (40). The adolescents with MDD showed reduced posterior alpha compared to HCs, and posterior alpha activity was related to depression symptoms, anhedonia symptoms, rumination (41). Microstate class B was significantly correlated with blood oxygenation level dependent (BOLD) changes in the striatum, extra-striatal cortex, and bilateral occipital cortex, which is related to the visual network (20, 42, 43). Microstate B was also found to be correlated to posterior temporal gyrus (44). Previous fMRI research of depressed subjects has found lower functional anterior cingulate cortex and posterior superior temporal gyrus connectivity compared to HCs (45). These regions play vital roles in integrating, collecting and processing information from the external environment and the internal body (46), and understanding emotions or feeling of other people (47). The increase of microstate B among MDD patients in the current study is in line with that MDD patients have deficits in cognitive function and depression symptoms, anhedonia symptoms. The higher occurrence of microstate B was mainly caused by the increased transition from “C to B” among MDD patients. Microstate C reflects part of the DMN, which is a task negative network (20). Among patients with MDD, overactivation of the DMN may be related to negative rumination (12). Other researchers have suggested that abnormalities in DMN connectivity are associated with deficits in emotion regulation among MDD patients (48). Therefore, the more frequent transition from “C to B” may reflect that adolescents with MDD have more frequent spontaneous rumination and emotion regulation.

Our results agree with previous studies reporting a decrease in microstate D (contribution and occurrence) among the adults MDD patients (22). Microstate D was found to be closely associated to the dorsal attention network (25). The decrease in microstate D is consistent with a large number of previous fMRI studies which showed decreased connectivity of the dorsal attention network among patients with MDD (11). The dorsal attention network is involved in internally- or externally-oriented attention. Decreases in connectivity of the dorsal attention network may predict deficits in attention among MDD patients (49). In addition, connectivity between the frontoparietal network and dorsal attention network is weaker among adults MDD patients than HCs (11). Decreased connectivity of the frontoparietal network and dorsal attention network is reported to be associated with higher levels of maladaptive rumination (50). Imbalances in these different network connections could lead to not only cognitive and executive dysfunction, but also emotional regulation dysfunction, which are characteristics of MDD (51).

The current study was the first study to explore the EEG microstate changes among adolescents with first episode MDD and HCs under resting-state. Although the participants in our study was adolescents, the results were partially consistent with previous studies among adults (22, 29). Both the adolescents and adults with MDD showed an increased (decreased) occurrence and contribution of microstate B (D). There are also some different findings among adults MDD patients. For example, Murphy et al. (22) found decreased duration of microstate D while we didn't. The differences may be explained by different methodologies, such as different clustering methods and different numbers of selected topographic maps (21, 22). Moreover, all subjects in this study are teenagers and their brains are in a stage of continuous development. It has been found in previous study that the duration of microstates increases continuously with increases in age (52). One thing that cannot be ignored is that depression itself may have effect on the brain development (53), resulting in divergence at different stages. One advantage of this study is that we assessed differences between the adolescents with first episode MDD and HC groups in the general resting condition, i.e., there is no cognitive task or emotional processing, so some task-related confounders can be eliminated (54). In addition, research conducted among adolescents may have a certain predictive ability for their mental health in adulthood.

## Limitations

There are several limitations of this study that should be noted. First, the EEG microstate depends on source modeling technology. However, the source modeling is based on the poor spatial resolution of EEG. Secondly, the patients with MDD included in the present study are adolescents. As the first depressive episode in early adolescence may be a manifestation of a later diagnosis of bipolar disorder, some of the patients in this study may be diagnosed with bipolar disorder in later life. Thirdly, the present study only included 35 patients and 35 HCs. A larger number of subjects should be included in future studies. Lastly, although there was no gender difference between the two groups, it should be pointed out that the gender of subjects was not completely matched between groups. There are differences in EEG microstates between males and females in terms of the duration and occurrence of specific microstates (52). Therefore, future research should include more subjects to study potential gender effects.

## Conclusions

The results of this study supported changes in microstate B and D of adolescents with MDD compared to HCs. It provided new insights into dynamic changes in resting-state EEGs of MDD adolescents, and provides some evidence for further exploration of biomarkers and early diagnosis of MDD among adolescents.

## Data Availability Statement

The datasets used and/or analyzed during the current study are available from the corresponding author upon reasonable request.

## Ethics Statement

The studies involving human participants were reviewed and approved by National Clinical Center Medical Ethics Committee of the Second Xiangya Hospital, Central South University. Written informed consent to participate in this study was provided by the participants' legal guardian/next of kin.

## Author Contributions

YQH, QTY, TYY, YRZ, XPG, CXH, and XRL contributed to conception and design of the study. YQH, QTY, TYY, YRZ, KZ, XYJ, and SXW participated in data collection and investigation and evaluation of the study. YQH, QTY, and XLC performed the statistical analysis. YQH and XLC wrote the first draft of the manuscript. XLC and XRL contributed to critical revision. All authors contributed to manuscript revision, read, and approved the submitted version.

## Funding

This study was supported by the National Key Research and Development Program of China (No. 2017YFC1309904), Hunan Provincial Innovation Foundation for Post-graduates (No. CX2019159).

## Conflict of Interest

The authors declare that the research was conducted in the absence of any commercial or financial relationships that could be construed as a potential conflict of interest.

## Publisher's Note

All claims expressed in this article are solely those of the authors and do not necessarily represent those of their affiliated organizations, or those of the publisher, the editors and the reviewers. Any product that may be evaluated in this article, or claim that may be made by its manufacturer, is not guaranteed or endorsed by the publisher.
